# CYP3A4, CYP2C9 and CYP2B6 expression and ifosfamide turnover in breast cancer tissue microsomes

**DOI:** 10.1038/sj.bjc.6601492

**Published:** 2004-02-17

**Authors:** R Schmidt, F Baumann, H Knüpfer, M Brauckhoff, L-C Horn, M Schönfelder, U Köhler, R Preiss

**Affiliations:** 1 Institute of Clinical Pharmacology, University of Leipzig, Härtelstr. 16-18, D-04107 Leipzig, Germany; 2 Department of Neuroanatomy, Paul-Flechsig-Institute for Brain Research, University of Leipzig, Jahnallee 59, 04109 Leipzig, Germany; 3 Clinic of Surgery, Martin-Luther-University of Halle-Wittenberg, Ernst-Grube-Str. 40, 06120 Halle (Saale), Germany; 4 Institute of Pathology, University of Leipzig, Liebigstr. 26, 04103 Leipzig, Germany; 5 Clinic of Surgery I, University of Leipzig, Liebigstr. 20a, 04103 Leipzig, Germany; 6 Clinic of Gynecology and Obstetrics, Hospital St Georg, Delitzscher Str. 141, 04129 Leipzig, Germany

**Keywords:** cytochrome P450, breast cancer, ifosfamide turnover

## Abstract

Ifosfamide is a prodrug that requires bioactivation by cytochrome P450 for antitumour activity. Up to now, little is known, to what extent in addition to the liver the ifosfamide metabolism may occur intratumorally. For this purpose, we investigated the expression of CYP3A4, CYP2C9 and CYP2B6 in breast cancer tissue using Western Blotting. Ifosfamide turnover was determined by detection of metabolites of the ifosfamide 4-hydroxylation and *N*-dechloroethylation in tumour microsomal incubations using HPLC/UV and LC/MS. The results demonstrate that all mammary tumours (*n*=11) reveal CYP3A4 expression; contents varied from 0.5 to 63 pmol mg_protein_^−1^. CYP2C9 (*n*=9) was present in all tested breast tumour samples, too, while CYP2B6 (*n*=10) protein could not be detected. All measured breast cancer microsomes (*n*=4) showed an ifosfamide *N*-dechloroethylation capacity in the range from 0.04 to 0.21 pmol min^−1^ mg_protein_^−1^, while metabolites of the 4-hydroxylation could not be determined. In conclusion, the detected presence of CYP3A4 and CYP2C9 in breast tumours offers the possibility of intratumoral turnover of ifosfamide. For the first time in the literature, we could demonstrate a turnover of ifosfamide by microsomal preparations from human breast cancer tissue. A calculated modulation of intratumoral ifosfamide turnover could considerably influence its therapeutic efficiency.

Cytochrome P450 enzymes are of potential importance in the aetiology and treatment of breast cancer due to their involvement in the activation and/or degradation of carcinogens, steroid hormones as well as bioactivation of some widely used cytostatic prodrugs like oxazaphosphorines. The metabolic activation of oxazaphosphorines is thought to take place primarily in the liver. However, the 4-hydroxylation products of oxazaphosphorines are characterised by a rapid half-life and their spontaneously decomposed and tumourstatic active phosphoramide mustards show a low membrane permeability (Lenssen and Hohorst, 1979; Patterson and Murray, 2002). It is assumed that an intratumorally occurring oxazaphosphorine bioactivation, in addition to the liver, could be important in the realisation of their cytotoxic effects. According to Patterson and Murray (2002), phenotyping of patient tumours for CYPs 2B, 2C and 3A prior to oxazaphosphorine therapy may help predict clinical outcome.

Ifosfamide, a member of the oxazaphosphorine class, requires P450-mediated bioactivation via 4-hydroxylation to form cytotoxic metabolites, whereas a CYP-dependent *N*-dechloroethylation reaction produces inactive ifosfamide metabolites. Principal catalysts of the hepatic ifosfamide 4-hydroxylation are CYP3A4 and CYP2C9 enzymes, while CYP3A4 and CYP2B6 have demonstrated the major isoforms in the hepatic *N*-dechloroethylation reaction (Chang *et al*, 1993, Roy *et al*, 1999a,1999b, Preiss *et al*, 2002).

In human breast tumours, CYP3A protein, the most interesting with regard to ifosfamide metabolism, could be detected, but its reported expression rate is highly variable and dependent on the detection method used. In immunohistochemical investigations, detected CYP3A in breast tumour tissue varied from 0 to 100% (Forrester *et al*, 1990; Murray *et al*, 1993; Yokose *et al*, 1999). Using Western Blotting methods, Chabot *et al* (1996) showed the presence of CYP3A4 in all 10 tested breast cancer samples, while Forrester *et al* (1990), Albin *et al* (1993) and Hellmold *et al* (1998) described negative results. Reverse transcription–PCR analysis demonstrated the presence of CYP3A4 mRNA in 15% (Huang *et al*, 1996) and 75% (Hellmold *et al*, 1998) of tested breast cancer samples, respectively.

The situation with regard to CYP2C9 expression is similarly contradictory. Detection rates of CYP2C protein by Western Blotting varied from 0% (Albin *et al*, 1993) to 100% (Forrester *et al*, 1990), again, while reported rates using either anti-rat 2C-antibodies (Forrester *et al*, 1990) or antibodies raised against purified human CYP2C9 (recognising 2C8, 2C9, 2C18 and 2C19 on Western Blotting) (Yokose *et al*, 1999) in immunohistochemistry amounted to 33 and 39%, respectively. By RT–PCR analysis, a CYP2C fragment could be detected in all tested tumour tissue samples (Huang *et al*, 1996; Hellmold *et al*, 1998).

Two investigations on CYP2B6 expression in breast cancer demonstrated that CYP2B6 mRNA was expressed in breast cancer samples (Ìscan *et al*, 1998, Hellmold *et al*, 1998). No data exist about the expression of the CYP2B6 protein in this tumour.

Regardless of these contradictory results, fundamental conditions for possible ifosfamide turnover seem to be present in human breast cancer tissue. However, the identification of various CYP mRNA and protein in tumours does not provide evidence of enzyme activity. It is essential that if these tumoral CYP forms are to be a focus for P450-directed prodrug development, then the intrinsic activity of the isoforms identified must be demonstrated (Patterson *et al*, 1999).

Therefore, we firstly, for our knowledge, investigated the possibility of intratumoral ifosfamide turnover in human breast cancer *in vitro* using microsomal preparations from breast cancer tissue and highly sensitive HPLC/UV and LC/MS detection to determine metabolites of ifosfamide bioactivation (4-hydroxylation) and *N*-dechloroethylation as well as Western Blotting to detect the expression of CYP3A4, CYP2C9 and CYP2B6 in the tumour samples.

## MATERIAL AND METHODS

### Chemicals

Ifosfamide and its metabolites were a gift from Asta Medica AG (Frankfurt, Germany). Acrolein, mesna, chloroacetaldehyde, 2,4-dinitrophenylhydrazine, glucose-6-phosphate and NADPH were purchased from Sigma-Aldrich Chemie GmbH (Steinheim, Germany). Glucose-6-phosphate dehydrogenase from yeast and NADP were from Boehringer Mannheim GmbH (Mannheim, Germany).

Anti-human P450 3A4 antibodies were purchased from Oxford Biomedical Research, (Oxford, USA). Anti-human CYP3A5 (WB-3A5), anti-human CYP2C9 (WB-2C9) and anti-human CYP2B6 (WB-2B6) Western Blotting kits as well as recombinant human CYP3A4, CYP3A5, CYP3A7, CYP2C9^*^1, CYP2B6 and human NADPH P450-reductase were purchased from Gentest Corporation (Woburn, USA). ECL-reagents and ECL-hyperfilm were from Amersham Buchler GmbH & Co KG (Braunschweig, Germany).

Solvents were HPLC grade from JT Baker BV (Deventer, Holland). All other chemicals not listed were purchased in the highest purity available.

### Patients

The study was approved by the Leipzig University Ethics Committee, and written consent was obtained from each patient. The investigation was carried out with 11 breast cancer patients (aged 45–92 years). The tumoral tissue specimens removed at the time of surgery were promptly trimmed of fat and connecting tissues. A part of each tissue sample was reserved for histopathological examination and the remainder was rapidly frozen in liquid nitrogen and stored at −70°C.

### Preparation of human breast cancer tissue microsomes

Frozen tissue was homogenised in phosphate buffer (pH 7.4, 1/15 M potassium phosphate, 1 mM EDTA), the homogenate centrifuged at 11 000 **g** and 2°C for 30 min and the resultant supernatant subjected to centrifugation at 100 000 **g** and 2°C for 60 min. The resulting microsomal pellet was resuspended in phosphate buffer containing 0.25 M sucrose and stored at −70°C until use. Protein concentrations were measured according to the method of Bradford (1976).

### Immunoblotting and CYP isoenzyme quantitation

Immunochemical determination was performed using several commercially available anti-human P450 3A4, 3A5, 2C9 and 2B6 antibodies and microsome preparations from human lymphoblastoid cell lines transfected with human P450 isoenzyme cDNA as standards.

Briefly, microsomal proteins and standards were separated electrophoretically on 12% resolving polyacrylamide mini-gels using a Mini Protean II electrophoresis unit (Bio-Rad Laboratories GmbH München, Germany) and then quantitatively transferred onto nitrocellulose sheets (0.45 *μ*m). After blocking for 1 h in TRIS-buffered saline containing 5% (w v^−1^) membrane blocking reagent, the membranes were incubated in diluted primary antibody for 1 h. The sheets were then incubated in diluted biotinylated secondary antibody for 1 h followed by incubation of the blots in diluted streptavidin-horseradish-peroxidase for 20 min. ECL-reagents and ECL-hyperfilm were used for detection. In the case of CYP3A4, the calibration curve was calculated from five concentrations of the relevant standard on each blot. The intensity of the CYP3A4 staining was quantitated using a ScanJet 4c scanner (Hewlett Packard) and the Sigma Gel software from Jandel Scientific.

### Microsomal incubations

Microsomal incubations from human breast cancer tissue samples were prepared according to the method of Ruzicka and Ruenitz (1992) with minor modifications. Mixtures contained per ml 1 mg microsomal protein, potassium phosphate buffer, pH 7.4, 40 *μ*mol, potassium chloride 120 *μ*mol, magnesium chloride 5 *μ*mol, mesna 300 *μ*g, either 2 *μ*mol NADPH or NADP 0.4 *μ*mol, glucose-6-phosphate 120 *μ*mol and glucose-6-phosphate dehydrogenase 0.6 U.

After prewarming in a 37°C shaking water bath for 10 min in the presence of the NADPH-generating system or NADPH, the reaction was initiated by adding ifosfamide (dissolved in 1.15% potassium chloride) in a final concentration of 500 *μ*g ml^−1^ (≅2 mM). The incubation time was 4 h. All investigations included control incubations without microsomes.

Aliquots of every sample were withdrawn at 0 min (after 10 min preincubation) and 4 h. The reaction was stopped by freezing in liquid nitrogen and the samples were stored at −70°C until use.

### Ifosfamide 4-hydroxylation

The 4-hydroxylation of ifosfamide was measured using an HPLC/UV method to quantify the liberated acrolein. After removal of protein by addition of 200 *μ*l 5% ZnSO_4_ and 200 *μ*l 2.5% Ba(OH)_2_ ml^−1^ and following centrifugation at 450 **g** for 10 min at 4°C, 500 *μ*l of solution was mixed with 100 *μ*l dinitrophenylhydrazine (1.56 mg ml^−1^ dissolved in 2 N HCl) and 100 *μ*l chloroacetaldehyde and incubated for 10 min at room temperature. After the derivatisation and extraction with 2 ml hexane, the aqueous phase was submitted to solid phase extraction. The following conditions were chosen for the chromatography: gradient – A: 20% acetonitrile/20 mM KH_2_PO_4_ 0.1% H_3_PO_4_; B: 80% acetonitrile/20 mM KH_2_PO_4_ 0.1% H_3_PO_4_; scheme: 0–10 min A, 10–35 min A → B, 35–38 min B → A, 38–60 min A; flow 1 ml min^−1^, column Nucleosil 100 RP18 (5 *μ*m). Daily calibration curves were used. The detection wavelength was 357 nm. The detection limit was approximately 20 ng ml^−1^.

### Ifosfamide *N*-dechloroethylation

The *N*-dechloroethylation of ifosfamide was measured using a high-sensitive HPLC/MS method to quantify the liberated 2- and 3-dechloroethylifosfamide.

A measure of 500 *μ*l of the microsomal incubation was mixed with 150 *μ*l 0.1 N NaOH. After double extraction with 2 ml aliquots of chloroform and evaporation with nitrogen, the residues were resolved in 100 *μ*l 30% methanol/20 mM ammonium acetate with ketocyclophosphamide (1 *μ*g) as internal standard. Detection was performed using an SSQ 7000 mass spectrometer (Finnigan), a Consta Metric 4100 MS HPLC pump (TSP) and a Spectra System AS 3000 autosampler (TSP) with an Ultrasep ES 100 Pharm (SEPSERV) RP 18 (125 mm × 2 mm; 5 *μ*m) column. The mobile phase consisted of 20% methanol and 80% 20 mM ammonium acetate containing 1 ml acetic acid per litre at a flow of 0.3 ml min^−1^. The injection volume was 20 *μ*l. The mass spectrometry parameters were: 10 V CID-positive ionisation, temperature of capillary 220°C and a needle voltage of 4.5 kV. We used SIM-modes 199 and 275 for 2- and 3-dechloroethylifosfamide, and ketocyclophosphamide. The detection limit was approximately 2 ng ml^−1^.

## RESULTS

### Histopathological examination of breast tumour specimens

**Figure 1:**
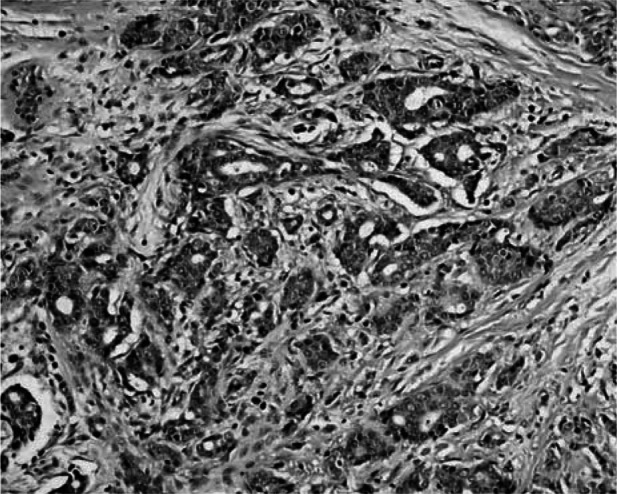
Example of histopathological examination of breast tumour specimen (invasive ductal carcinome, grade 2, breast cancer sample Eli 8).

Histopathological examination of breast tumours revealed an infiltrating ductal carcinoma in eight cases (for example Figure 1

), in one case each a ductal/lobular and lobular carcinoma and one ulcerated tumour. The histological gradation mainly showed grade G2 and G3.

### Western Blotting of CYP3A4, CYP2C9 and CYP2B6 in breast cancer microsomes

**Figure 2:**
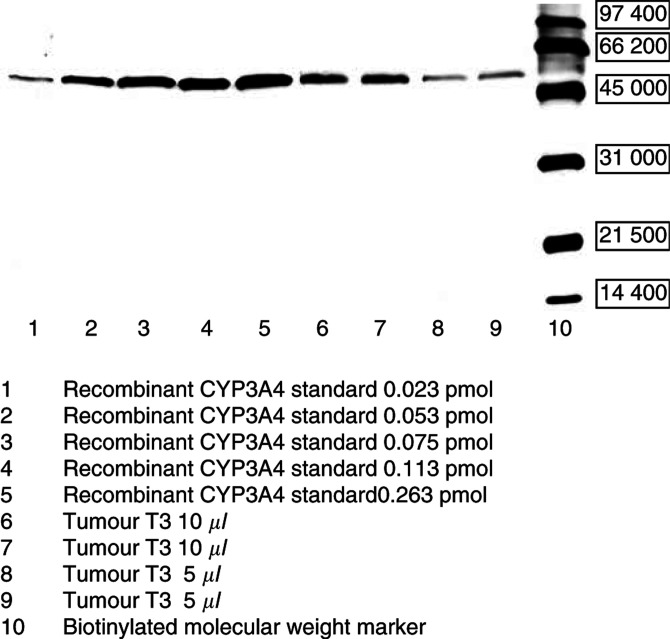
Example of CYP3A4 Western blot (breast cancer sample T3). All mammary tumours (*n*=11) reveal CYP3A4 expression.

CYP3A4 was detected in 11 out of 11 microsomal preparations. An example of CYP3A4 quantitation using five concentrations of recombinant CYP3A4 standard is given in Figure 2

.

**Figure 3:**
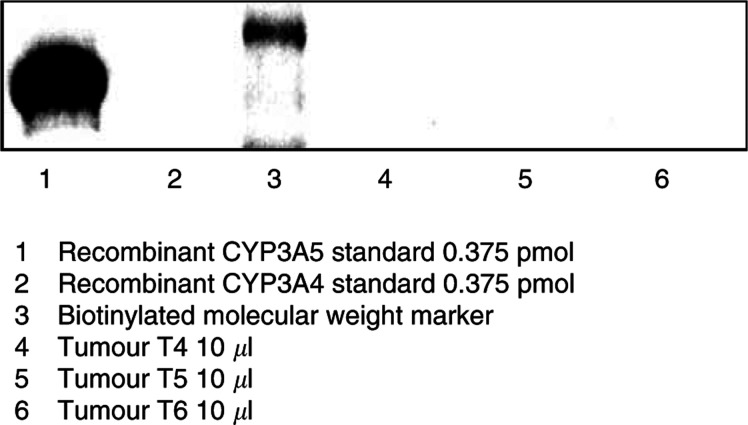
Anti-human CYP3A5 Western blot. None of the breast samples tested (10 out of 11) showed CYP3A5 immunoreactivity.

In contrast, none of the samples tested (10 out of 11) showed CYP3A5 immunoreactivity (for example Figure 3

).

**Figure 4:**
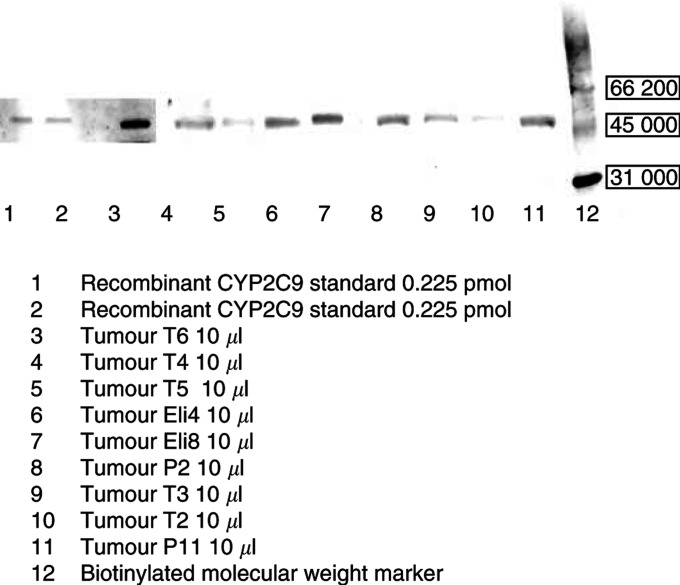
Anti-human CYP2C9 Western blot. CYP2C9 was present in all tested breast tumour samples (nine out of 11).

**Figure 5:**
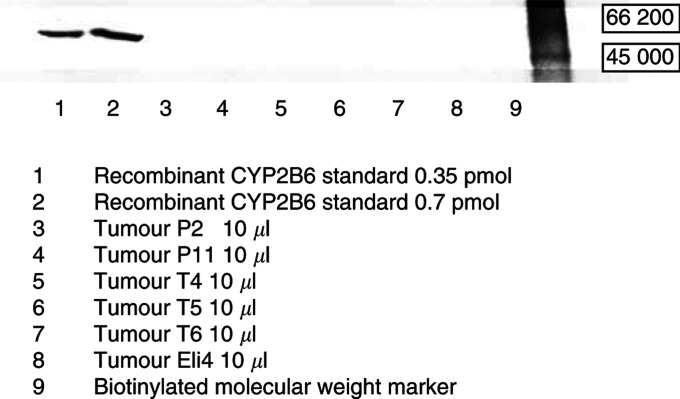
Anti-human CYP2B6 Western blot. CYP2B6 protein could not be detected in all tested breast tumours (10 out of 11).

All investigated breast tumours (nine out of 11) revealed low but nevertheless positive staining of the CYP2C9 protein (Figure 4

). CYP2B6 protein was not expressed in any tumour sample tested (10/11; for example Figure 5

). The results of all CYP Western Blots are summarised in Table 1

**Table 1:** CYP3A4/5, CYP2C9 and CYP2B6 contents in breast cancer samples

**Tumour sample**	**CYP3A4 (pmol mg_protein_^−1^)**	**CYP3A5**	**CYP2C9**	**CYP2B6**
T2	0.5	−	+	−
T3	12	−	+	−
T4	53	−	+	−
T5	18	−	+	−
T6	38	−	+	−
E1	0.5	n.m.	n.m.	n.m.
Eli 3	6	−	n.m.	−
Eli 4	15	−	+	−
Eli 8	63	−	+	−
P 2	7	−	+	−
P 11	6	−	+	−

n.m.=not measured.

.

### Ifosfamide 4-hydroxylation

Acrolein could not be detected in all tested breast cancer samples T2, T3, T4 and Eli3.

### Ifosfamide *N*-dechloroethylation

In a preliminary examination, we tested the direct addition of NADPH to the microsomal incubations. No measurable ifosfamide *N*-dechloroethylation activity could be detected in the tested four breast cancer samples T3, E1, Eli4 and Eli3. Using the addition of NADPH-generating system to the microsomal incubations, ifosfamide *N*-dechloroethylation activity of the tested four breast cancer samples T4, T5, T6 and Eli8, measured as sum from 2- and 3-dechloroethylifosfamide, ranged from 0.04 to 0.21 pmol min^−1^ mg_protein_^−1^ (mean: 0.12±0.07 pmol min^−1^ mg_protein_^−1^).

Ifosfamide *N*-dechloroethylation activities of the four tested tumour samples T5, T6, P2 and P11 with the addition of NADPH-generating system+5 U ml^−1^ P450-reductase were in the range from 0.07 to 1.46 pmol min^−1^ mg_protein_^−1^ (Table 2

**Table 2:** Ifosfamide *N*-dechloroethylation activity (sum from 2- and 3-dechloroethylifosfamide) with addition of NADPH-generating system and NADPH-generating system+P450-reductase

**NADPH generating system**		**NADPH generating system+P450-reductase**	
**Tumour sample**	***N*-dechloroethylation (pmol min^−1^ mg_protein_^−1^)**	**Tumour sample**	***N*-dechloroethylation (pmol min^−1^ mg_protein_^−1^)**
T4	0.04		
T5	0.21	T5	0.07
T6	0.12	T6	1.46
Eli8	0.11		
		P2	0.25
		P11	0.07

). Owing to a very limited quantity of tumoral microsomes, a parallel measurement of basic ifosfamide *N*-dechloroethylation and with addition of P450-reductase was practicable in two cases (T5, T6) only.

## DISCUSSION

Investigation of the extrahepatic, especially intratumoral, presence, function and regulation of cytochrome P450 has become a major area of interest in recent years. P450 enzymes have been detected in human breast cancers, but data are conflicting. Knowledge of their capacity to bioactivate cytostatic prodrugs like oxazaphosphorines is very scant.

Therefore, we firstly investigated the possibility of ifosfamide turnover in human breast cancer microsomes using highly sensitive HPLC/UV and LC/MS detection methods to determine ifosfamide metabolites and Western Blotting to detect the expression of CYP3A4, CYP2C9 and CYP2B6 proteins in the tumours.

**Figure 6:**
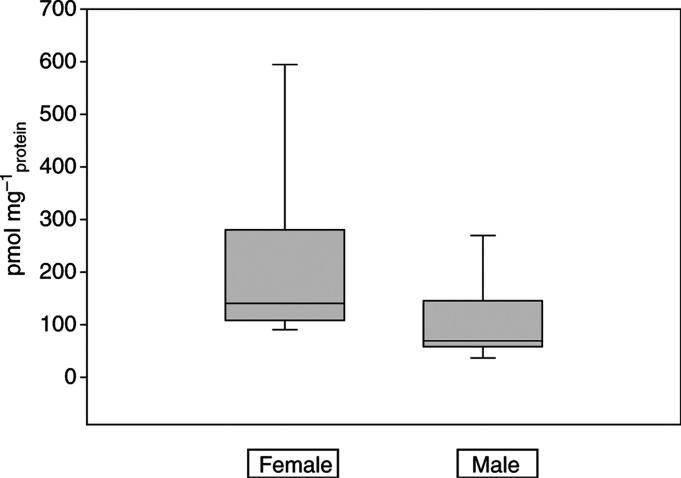
Comparison of CYP3A4 contents in female and male liver microsomes. Box plots present the median, 5th, 25th, 75th and 95th percentiles.

Using a polyclonal anti-human CYP3A4 antibody (detecting CYP34A, 3A5 and 3A7) and recombinant CYP3A4 as a standard, we detected and quantified CYP3A by Western Blotting in all 11 tested breast cancer samples. The content of this isoform varied from 0.5 to 63 pmol mg_protein_^−1^ (19.9±21.6 pmol mg_protein_^−1^). Comparatively, the same antibody detected levels of CYP3A in female liver microsomes, which has been demonstrated to be significantly higher than in male samples, amounting to 141 and 69 pmol mg_protein_^−1^ (median; *P*=0.038, Mann–Whitney rank sum test), respectively (Figure 6

) (Schmidt *et al*, 2001). The use of a CYP3A5-specific antibody allowed us to exclude the expression of CYP3A5 in the tested tumours, but the involvement of CYP3A7 could not be further clarified because of the absence of a specific anti-CYP3A7 antibody.

Using a monoclonal anti-human CYP3A4 antibody, Chabot *et al* (1996) also showed the presence of CYP3A protein in all 10 tested breast cancer samples. In the investigations of Chabot *et al* (1996), tumour CYP3A4 levels were found to be more than 120-fold lower than in corresponding liver samples. These relatively low tumoral P450 levels could be explained by the fact that both our investigation and the study of Chabot *et al* (1996) exclusively used the microsomal fraction, in accordance with most groups working in this area. However, tumour cells, in contrast to liver, have a paucity of smooth endoplasmic reticulum and the usual microsomal preparation may not be suitable in the case of tumour tissue. Furthermore, tumoral P450 may not be exclusively located in the smooth endoplasmic reticulum (Murray, 2000). Examination of other subcellular fractions including whole tumour homogenate should be included in future determination of tumour CYP content.

In this study, firstly using a specific anti-human CYP2C9 antibody in Western Blotting, which does not crossreact with other CYP2C-members (CYP2C8, CYP2C18, CYP2C19), we could also show weak expression of CYP2C9 protein in all human breast tumours tested. In contrast, Albin *et al* (1993) and Hellmold *et al* (1998), using anti-human CYP2C8-10 and anti-human CYP2C10, respectively, did not detect CYP2C protein in breast cancer tissue by this method.

Additionally, CYP2B6 detection with an antibody strongly reacting with human cDNA-expressed CYP2B6 (detection limit approximately 0.2 pmol) showed no positive immunostaining in all tested breast tumour samples (10 out of 11). Assuming a proportion of CYP2B6 similar to the human liver, where in our previous investigation (Preiss *et al*, 2002) the percentage of this P450 isoform amounted to only 2.6% of total P450, the sensitivity of the used Western Blotting is not enough to detect the minor isoenzyme CYP2B6.

However, our results demonstrate that the investigated breast cancer samples express at least two members of the CYP family, raising the possibility that CYP3A4- and CYP2C9-catalysed metabolism in tumour tissue could result in local bioactivation and/or deactivating *N*-dechloroethylation of ifosfamide.

In fact, all measured breast cancer samples (*n*=4) showed minimal ifosfamide *N*-dechloroethylation activities *in vitro* (0.12±0.07 pmol min^−1^ mg_protein_^−1^), whereas metabolites of ifosfamide 4-hydroxylation (*n*=4) could not be detected. However, it should be taken into account that in our investigations the detection limit of the ifosfamide 4-hydroxylation reaction was 10-fold higher than that of ifosfamide *N*-dechloroethylation reaction (20 *vs* 2 ng ml^−1^). In comparison, our previous investigations on 10 human female liver samples showed activities of 132±57 pmol min^−1^mg^−1^ protein for ifosfamide *N*-dechloroethylation and 1143±208 pmol min^−1^ mg^−1^ protein for ifosfamide 4-hydroxylation.

Only a limited number of comparable investigations exist that analysed the metabolic capacity of human breast tumour tissue such as the both CYP1A1-mediated ethoxyresorufin *O*-deethylase (Ìscan *et al*, 1999) and aryl hydrocarbon hydroxylase activities (Pyykko *et al*, 1991).

Senler *et al* (1985) found the average cyclohexane (as a substrate for several forms of cytochrome P450) hydroxylase activity in 139 human breast tumour microsome preparations in the range of those observed for untreated rabbit liver microsomes.

In contrast, the catalytic activity of the CYP2A6-specific coumarin hydroxylation varied in four measured breast cancer microsomes between 1.6 and 4 pmol min^−1^ mg_protein_^−1^, whereas in human liver microsomes this activity was approximately 24 nmol min^−1^ mg_protein_^−1^ (Hellmold *et al*, 1998). Thus, Hellmold *et al* (1998) found a similar breast tumour/liver activity ratio for coumarin hydroxylation as we did for ifosfamide *N*-dechloroethylation.

The NADPH cytochrome P450-reductase is an obligatory and often rate-limiting component of microsomal P450-dependent activities. P450-reductase and CYP2B6 gene cotransferred into gliosarcoma cells significantly increased the cyclophosphamide and ifosfamide activation (Jounaidi *et al*, 1998). Sequeira and Strobel (1996) demonstrated on rat brain microsomes an increased imipramine metabolism by addition of exogenous purified P450-reductase. Therefore, to test whether the measured minimal ifosfamide *N*-dechloroethylation activity could be improved by addition of this cofactor, P450-reductase (5 units ml^−1^) was added. Owing to the very limited quantities of breast tumour microsomes, a parallel measurement of basic ifosfamide *N*-dechloroethylation and ifosfamide *N*-dechloroethylation after addition of P450-reductase was practicable in two cases only. Our results demonstrated an enhancement of ifosfamide *N*-dechloroethylation activity in the tumour microsomal preparation T6, while with the addition of P450-reductase this activity was decreased in tumour microsomes T5. Possibly, this discrepancy can be explained by the use of different lots of P450-reductase. However, further investigations are needed to clarify the importance of this cofactor in tumoral CYP-mediated metabolism.

In conclusion, the presence of two important ifosfamide catalysts CYP3A4 and CYP2C9 in breast tumours suggests that its local turnover could be of importance in cancer therapy. We succeeded in detecting minimal ifosfamide *N*-dechloroethylation activities (0.12±0.07 pmol min^−1^ mg_protein_^−1^) using LC/MS in all measured breast cancer microsomes, while the HPLC/UV method developed was not sensitive enough to detect metabolites of the ifosfamide 4-hydroxylation. This is the first demonstration of the ifosfamide turnover by microsomal preparations from human breast cancer tissue. Finally, expression of xenobiotic metabolising enzymes with actual basic metabolic capacity in breast tumours has been identified as a potentially important factor in the modulation of the, so far underestimated, intratumoral CYP-mediated prodrug activation.
